# Investigating water/oil interfaces with opto-thermophoresis

**DOI:** 10.1038/s41467-022-31546-3

**Published:** 2022-06-29

**Authors:** Youngsun Kim, Hongru Ding, Yuebing Zheng

**Affiliations:** 1 Materials Science and Engineering Program and Texas Materials Institute, The University of Texas at Austin, Austin, TX 78712 USA; 2 Walker Department of Mechanical Engineering, The University of Texas at Austin, Austin, TX 78712 USA

**Keywords:** Applied physics, Optical manipulation and tweezers, Soft materials, Surfaces, interfaces and thin films

## Abstract

Charging of interfaces between water and hydrophobic media is a mysterious feature whose nature and origin have been under debate. Here, we investigate the fundamentals of the interfacial behaviors of water by employing opto-thermophoretic tweezers to study temperature-gradient-induced perturbation of dipole arrangement at water/oil interfaces. With surfactant-free perfluoropentane-in-water emulsions as a model interface, additional polar organic solvents are introduced to systematically modify the structural aspects of the interface. Through our experimental measurements on the thermophoretic behaviors of oil droplets under a light-generated temperature gradient, in combination with theoretical analysis, we propose that water molecules and mobile negative charges are present at the water/oil interfaces with specific dipole arrangement to hydrate oil droplets, and that this arrangement is highly susceptible to the thermal perturbation due to the mobility of the negative charges. These findings suggest a potential of opto-thermophoresis in probing aqueous interfaces and could enrich understanding of the interfacial behaviors of water.

## Abstract (web summary)

Charging of interfaces between water and hydrophobic media is a mysterious feature whose nature and origin remain under debate. Here, the authors use opto-thermophoretic tweezers to investigate the interfacial behavior at water/oil interfaces in terms of thermal perturbation of dipole arrangement.

## Introduction

Water is the most abundant substance within the human body and on the Earth’s surface, which is essential in the ecosystem and industry. The major characteristics that make water such a special liquid are its high polarity and strong hydrogen bonds, which enable, for example, hydration and transport of various molecules. Despite its irreplaceable use in our daily lives, ironically, water remains a conundrum in part. One of the mysterious phenomena is the spontaneous charging of interfaces between water and nonpolar media. Water/oil and water/air interfaces were observed to be negatively charged in electrokinetic measurements^1–4^. Efforts have been made to unravel the origin of negative charging at the direct contact of two immiscible phases. pH-dependence of electrokinetic properties, i.e., electrophoretic mobility and zeta potential, has been primarily studied, which suggests the preferential adsorption of autoionized hydroxide ions (OH^−^) at the interface^1–3,5^. Spectroscopic methods such as second-harmonic generation (SHG) and vibrational sum frequency generation (SFG) have been employed to acquire molecular information. However, many spectroscopic results and interpretations along with computational work are not consistent with one another and a consensus on the nature and origin of interfacial charging has not been reached^6^. So far, the interfacial charging has been attributed to OH^−^ adsorption^7^, H_3_O^+^ adsorption^8^, imbalanced hydrogen bonding and charge transfer without autoionized species^9,10^, or the presence of acid impurities^11^. Analytical methods are needed for a better understanding of water’s interfacial behaviors, which can further elucidate the various roles of water at interfaces present in biological and chemical systems (e.g., cell membrane dynamics and function^12–14^, protein folding^15,16^, and electrochemical reactions^17,18^).

We believe that a combination of improved analytical models and techniques is necessary to overcome the inconsistency and eventually depict a real picture of the interface. Historically, surfactant-free oil-in-water emulsion droplets, which are microscale or nanoscale in size, have been widely chosen as models for the interfacial study as they provide neat biphasic interfaces^9,19–21^. Colloids are stabilized by repulsive potentials, mainly electrostatic and/or steric contributions by surfactants that prevent agglomeration upon collision. In surfactant-free oil-in-water emulsions that contain liquid molecules and ion species in majority, a possible scenario of the colloidal stabilization is narrowed down to the surfactant-like role of these species at the interface. Therefore, liquid molecules and ions at the interface are anticipated to have certain structures different from the bulk phase to behave as surfactants. In pH-dependent electrokinetic measurements, the addition of acid or base results in an increase in ionic strength of an overall sample, making it hard to exclude the effects of counterions on the interfacial charges and structures. In the spectroscopic methods, pulsed light sources (IR and 800 nm) can affect the interfaces during the measurements^22^. Errors can also occur in deconvolution of the spectra, resulting in ambiguous molecular information for the interfaces^6^.

**Fig. 1:**
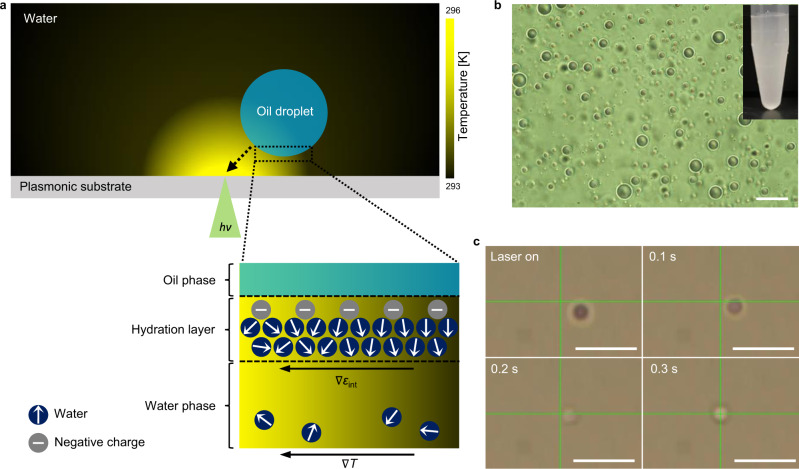
Working principle of OTTs and trapping of a PFP droplet. **a** A temperature gradient is created by the photothermal conversion of light at a plasmonic substrate, and a water-dispersed oil droplet migrates to a hot region. Water molecules and negative charges are arranged at the water/oil interface, having a radial orientation of water dipoles (indicated by arrows). The temperature gradient (∇*T* = d*T/*d*r, r*: distance from the laser beam) induces a permittivity gradient (∇*ε*_int_*)* along the interfacial water layer by thermal perturbation of interfacial water ordering, leading to a thermophoretic migration of the droplet toward the hot region. **b** Optical image of PFP droplets dispersed in water (scale bar: 10 μm). Inset is a photograph of PFP-in-water emulsions. **c** Optical images of a PFP droplet under an optical input in the course of time (scale bars: 10 μm). Crossbars indicate the position of the laser beam. The droplet is trapped once it is close to the laser beam.

Here, we seek to better elucidate the interfacial charging phenomenon under a thermal field and variable solvent environments. We hypothesize that the interfacial charging stems from specific structuring of water and is related to the dielectric property and hydrogen bonding of interfacial water and/or ionized species. In this regard, a local change in temperature along the interface can alter the structure-related dielectric property of interfacial water. In addition, polar organic solvents can interfere with the hydrogen bond network of interfacial water to cause another mode of interfacial perturbation. To systematically study the effects of these variables on the interfacial water, we employ opto-thermophoretic tweezers (OTTs) to analyze surfactant-free perfluoropentane (PFP)-in-water emulsions as a model. OTTs operate under a microscale thermal field created by a laser beam irradiated on a photothermal substrate. On the charged surface of colloidal objects, water molecules are arranged with a radial dipole alignment by electrostatic interactions. The degree of dipole alignment and water confinement decreases with the distance from the surface, forming a so-called electrical double layer of strong and weak confinement regimes (Helmholtz and diffuse Gouy-Chapman layers, respectively). In water, the thickness of the Helmholtz layer is <1 nm on negative surfaces with hydrated protons as counterions^23^ and the Debye length is estimated to be a few hundred nm at pH 5.5-7.0 regarding low ionic strength^24^. Due to the ordering of water molecules, the dielectric constant of interfacial water in the double layer is distorted, which is much lower than that of bulk water^25,26^. Under a temperature gradient field, the degree of order of interfacial water is reduced by thermal perturbation, inducing a permittivity gradient in the double layer along the temperature gradient^27^. The thermally induced unbalance of the interfacial permittivity generates a slip flow along the interface, leading to the thermophoretic motion of the colloidal objects (Fig. 1a)^26,28,29^. With the capabilities of generating a local thermal field and revealing information of interfacial dipole arrangement, OTTs are well suited to investigate the interfacial charging. We choose PFP-in-water emulsions for a neat water/oil interface. PFP and other perfluorocarbon liquids are both superhydrophobic and lipophobic due to the strength of the C − F bond and resultant low intermolecular interactions^30,31^. The contribution of the oil phase to the interface is minimized with PFP, and we can focus on the interfacial behavior of the aqueous species. Moreover, the immiscibility of PFP with most of the organic solvents enables us to create diverse solvent environments with water-miscible solvents while keeping the oil phase intact.

## Results

### Opto-thermophoretic trapping of oil droplets

Surfactant-free metastable PFP-in-water emulsions were prepared by bath sonication (1.74 µm in average diameter, Fig. 1b). Diluted emulsions were placed under an optical microscope and a 532 nm laser beam (0.06 mW μm^−2^) was focused on a glass substrate covered by arrays of gold nanoislands (also known as plasmonic substrate) (see Supplementary Fig. 1 for an optical setup). As shown in Fig. 1c and Supplementary Movie 1, a droplet rapidly responds to a light incidence, being trapped at a focal point. As the laser beam position with respect to the sample is moved, the droplet follows, which demonstrates the capability of OTTs in performing liquid-in-liquid manipulation (see Supplementary Movie 2). Moreover, multiple droplets are assembled around a hot spot without merging (Supplementary Fig. 2) in a concentrated droplet sample, indicating that droplets are stabilized to the extent that they do not collapse when brought together. These trapping behaviors are in accordance with those of negatively charged colloidal objects under OTTs reported earlier (e.g., polystyrene beads, silica microparticles, and biological cells)^26,29,32^. The zeta potential of PFP droplets is −48.6 mV, which is similar to that of other water-dispersed oil droplets and air bubbles in literature^1–3,6^. As per the working principle of OTTs, it can be drawn that water molecules are arranged on the droplet surface with a radial dipole orientation and an effective negative surface charge. This arrangement is expected to hydrate and stabilize the droplet, which contributes to a positive permittivity gradient over temperature and results in a thermophoretic force that attracts the droplet to the hot spot (Fig. 1a). The permittivity of ordered interfacial water is much lower than that of bulk water due to the suppression of dipolar fluctuation^33,34^. Thus, the thermal perturbation of pre-ordered interfacial dipoles at the hot region leads to a higher permittivity and subsequently a higher electric energy density.

**Fig. 2:**
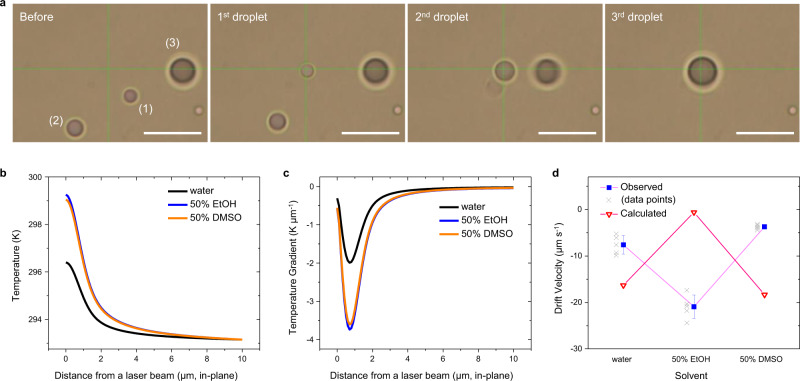
Trapping of droplets in different solvent compositions. **a** Optical images of PFP droplets in 50% EtOH under OTTs, which show merging of three droplets. Parenthesized numbers indicate the order of trapping. **b** Simulated in-plane temperature and **c** temperature gradient profiles from a laser beam in different solvent compositions: water (black), 50% EtOH (blue), and 50% DMSO (green). The negative sign of temperature gradient indicates the direction pointing toward the laser beam. **d** Observed (blue) and calculated (red) drift velocity of PFP droplets in different solvent compositions (mean ± standard deviation, *n* = 5). Gray symbols represent individual data points. Calculated values are obtained at fixed *τ* using Eq. (1) to assess the contribution of other available parameters (i.e., bulk solvent terms, temperature gradient, and zeta potential) to the drift velocity. The negative sign of the velocity indicates that the direction is the same as that of the temperature gradient. Source data are provided as a Source data file.

We further examined the trapping behaviors of PFP droplets in different solvent environments by adding polar organic solvents to water. It should be noted that emulsions were not formed when other organic solvents such as methanol, ethanol (EtOH), 2-isopropanol, acetone, acetonitrile, dimethyl formamide, and dimethyl sulfoxide (DMSO) completely replaced water (Supplementary Fig. 3). Thus, the stabilization of PFP droplets by interfacial charging is considered specific to water. In binary solvents (i.e., 50 wt% organic solvent + 50 wt% water), two opposite effects were observed: (i) rapid trapping and merging of trapped droplets for ethanol, methanol, 2-isopropanol, acetone, and acetonitrile as the organic solvents and (ii) weakened trapping for dimethyl sulfoxide and dimethyl formamide as the organic solvents. Figure 2a shows the merging of three droplets in 50% EtOH when trapped by OTTs at a hot spot. Incoming droplets coalesce swiftly into a pre-trapped droplet. For the further investigation, EtOH and DMSO were selected as representative organic solvents with the opposite effects due to availability of extensive literature on thermodynamics and other physical properties of their mixture with water. Although PFP has a partial solubility in EtOH (i.e., ~4.2 g PFP in 100 g EtOH), most of ethanol in the ternary liquid system used in our study, PFP − EtOH−water, is predicted to be extracted to the water phase. Given the limited solubility along with PFP − water immiscibility and EtOH−water miscibility, a ternary liquid-liquid phase diagram can be drawn as Supplementary Fig. 4 (type II mixture, similar to methanol-cyclohexane-water^35^). Thus, at the composition of 49 wt% water, 49 wt% EtOH, and 2 wt% PFP, a two-phase equilibrium is reached with two extremes of pure PFP and 50 wt% EtOH/water, considering the curvature of the binodal curve. While the size distribution of PFP droplets in water, 50% EtOH, and 50% DMSO does not vary significantly (Supplementary Fig. 5), the colloidal stability is dependent on the solvent composition: less stable in 50% EtOH and more stable in 50% DMSO compared to pure water (Supplementary Fig. 6). In this regard, the merging behavior in 50% EtOH is attributable to poor colloidal stability (Fig. 2a).

### Theoretical analysis

The trapping velocity or drift velocity of the droplets was measured as −7.6 ± 2.0, −20.9 ± 2.5, and −3.7 ± 0.9 μm/s in water, 50% EtOH, and 50% DMSO, respectively (see Methods and Supplementary Fig. 7 for details of velocity measurements). To interpret the variation in the trapping velocity, a theoretical analysis was conducted. A drift velocity of a charged particle under a temperature gradient in OTTs is formulated as^36,37^

1 $${{{{{\bf{u}}}}}}=-{D}_{{{{{{\rm{T}}}}}}}\nabla T=\frac{{\varepsilon }_{{{{{{\rm{b}}}}}}}}{2\eta T}\frac{2\kappa }{2\kappa +{\kappa }_{{{{{{\rm{p}}}}}}}}\left(1+\tau \right){\zeta }^{2}\nabla T$$

The equation consists of bulk solvent terms (i.e., dielectric constant *ε*_b_, viscosity *η*, and thermal conductivity *κ*) and particle/solvent interface terms (i.e., permittivity-related term $\tau =\partial ({{{{{\rm{ln}}}}}}{\varepsilon }_{{int}})/\partial ({{{{{\rm{ln}}}}}}T)$ where *ε*_int_ is the permittivity of interfacial solvent and *T* is temperature, and zeta potential *ζ*), all of which vary with solvent compositions and determine the thermophoretic mobility *D*_T_. *κ*_p_ is the thermal conductivity of the particle. Please note that *τ* represents the thermal responsiveness of the interfacial permittivity (i.e., the dipole alignment of interfacial solvent molecules), which is a key factor for the trapping, and has a positive value (~2) for water in an electrical double layer on the charged particle surface^27^, which is different from a negative bulk counterpart. At a higher temperature, interfacial water molecules become less confined by surface charge, resulting in a higher permittivity. Thus, *ε*_int_ has its maximum at the surface region of the droplet near the laser beam and minimum on the opposite side. The other factors (e.g., dispersion and electric forces) that contribute to the thermophoresis of the charged particle are excluded because their contributions to drift velocity are much smaller than the permittivity-gradient-driven force^29,38,39^. The temperature gradient ∇*T* was simulated using a heat transfer model in liquid with a fixed Gaussian heat influx (see Materials and Methods for simulation details). The heat influx was derived from thermal imaging by quadriwave shearing interferometry (see Supplementary Figs. 8–10 and Supplementary Table 1 for details). More information on the effects of dispersion force and Marangoni flow is available in Supplementary Notes 1 and 2, Supplementary Table 2, and Supplementary Fig. 11.

As shown in Fig. 2b, c, the spatial temperature gradient is generated from the maximum temperature at a laser beam position throughout a medium at room temperature. In water, its maximum is around −2 K/μm at 1 μm from the beam center (in-plane). The higher temperature and larger in-plane temperature gradients in 50% EtOH and 50% DMSO than those in water were obtained due to the reduced specific heat and thermal conductivity of the mixtures. Zeta potentials for 50% EtOH and 50% DMSO were measured as −13.9 and −74.3 mV, respectively. We kept *τ* as a constant of 2 to evaluate the contributions of the other experimentally observable parameters (i.e., bulk solvent terms, zeta potential, and temperature gradient) to the drift velocity. The maximum drift velocities are calculated as −16.3 (water), −0.6 (50% EtOH), and –18.3 μm/s (50% DMSO), where the negative sign indicates the thermophilic behavior of the droplets (Table 1). As shown in Fig. 2d, a reverse trend across the solvent compositions is obtained between calculated and observed values. The bulk solvent terms and zeta potential contribute to a reduction of the trapping velocity for 50% EtOH and an increase for 50% DMSO in the calculation. Thus, the deviation between the calculated and observed values should arise from our assumption that *τ* is a constant. Based on the experimental velocities, we derived *τ* as 106 and 0.15 (relative to that for water) for the 50% EtOH and 50% DMSO, respectively. Therefore, the interfacial dipole alignment is more vulnerable to the thermal perturbation in the presence of EtOH while DMSO makes a rather rigid interfacial structure, compared to a pure water environment.

**Table 1:** Physical parameters of solvents at 20 °C, calculated thermophoretic mobility and drift velocity at a constant *τ* of 2, and relative *τ* derived from the observed drift velocity.

Solvent	*ε*_b_	*η* [cP]	*κ* [W m^−1^ K^−1^]	*ζ* [mV]	*D*_T_ [µm^2^ K^−1^ s^−1^]	∇*T*^*a*^ [K µm^−1^]	u [µm s^−1^]	u_obs_ [µm s^−1^] (95% CI)^b^	*τ* (relative)^c^
water	80.1	1.00	0.598	−48.6	8.21	−1.99	−16.3	−7.6 (−9.4, −5.9)	1
50% EtOH	50.3	2.41	0.317	−13.9	1.69	−3.74	−0.6	−20.9 (−23.1, −18.7)	106
50% DMSO	75.5	3.46	0.327	−74.3	5.08	−3.61	−18.3	−3.7 (−4.5, −2.9)	0.15

^a^The maximum temperature gradient from Fig. 2c is used for ∇*T* and *κ*_p_ is 0.05 W m^−1^ K^−1^.

^b^Confidence intervals (CI) at 95% for the observed velocity.

^c^Relative *τ* was calculated from experimentally observed drift velocities and other physical parameters and was normalized to the value for water.

### Comparison between droplets and particles

**Fig. 3:**
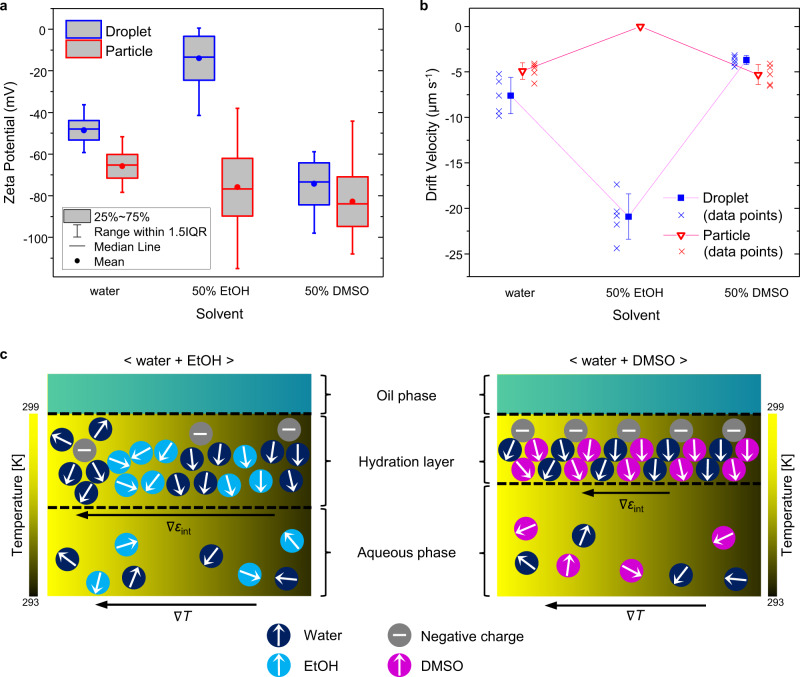
Comparison between droplet and particle systems and solvent-dependent scheme of the water/oil interface. **a** Zeta potential (obtained from zeta potential distributions, counts > 3 × 10^5^, error bars: 1.5 interquartile range (IQR)) and **b** drift velocity of PS beads (red) and PFP droplets (blue) (mean ± standard deviation, *n* = 5, cross symbols: individual data points). **c** Schematic illustration of the hydration layer in different solvent environments. EtOH destabilizes negative charges owing to weak dipole-charge interactions and weak hydrogen bonding with water, forming a diffuse, inhomogeneous, and therefore, thermally responsive interface. The strong complexation of DMSO with water constructs a rigid interface that reduces its thermal responsiveness. Source data are provided as a Source data file.

To better understand the solvent-dependent interfacial responses, we performed a comparison study of PFP droplets with carboxylate-functionalized polystyrene (PS) beads (2.19 μm in diameter). Figure 3a shows the zeta potentials of PS beads and PFP droplets. The higher potential of PS beads in the binary mixtures than that in water indicates the thicker electric double layers in the presence of polar organic solvents, given the fixed surface charge density and Henry’s function used in the measurements ($f\left(\kappa a\right) \sim 1.3$). Interestingly, beads in 50% EtOH are not trapped by OTTs while similar trapping velocities are observed for the beads in water and 50% DMSO (Fig. 3b). We estimated the relative *τ* as −0.5 (50% EtOH) and 3 (50% DMSO). These results are in sharp contrast to those from the droplets. The difference in the mobility of surface charges between beads and droplets can play a critical role in the different trapping behaviors. The negative charge of PS beads originates from surface-functionalized carboxylic acid by which an electric double layer is passively formed, whereas charging at the surfaces of PFP droplets is related to the specific coherent assembly of solvent molecules and negative charges required for the droplet stabilization. The charges on the droplet surfaces could be mobile. Therefore, the trapping behavior of PS beads is mainly governed by the effects of solvent properties. In contrast, the large variation of *τ* and trapping velocity in the droplet case, taken together with the deviation in the zeta potential, implies that the thermal stimulus affects both the solvent molecules and the charges at the interfaces. Consequently, an overall network of mobile negative charges and solvent molecules is regarded to behave as an interface that responds to the temperature gradient, suggesting a role of mobile charges in determining the interfacial permittivity change. The fluidic nature of the droplets and their interfaces without rigid surfactants implies that the droplets are more susceptible to fluctuation under an external electric field, which can contribute to a higher zeta potential than that of charged particles with the same surface charge density. It has been revealed that alcohols are immiscible with water at the microscale regime, forming separate clusters of water and alcohol that are responsible for their abnormally small mixing entropy^40^. For the PS bead case, the interfacial dipole arrangement at the interface is originally disordered to some extent in the presence of EtOH molecules due to intercalated EtOH domains and less accessible charge-dipole interactions, resulting in the higher permittivity of interfacial solvent molecules than that of pure interfacial water^34^. Under the temperature gradient, molecules at the hot region have a higher degree of freedom to be aligned with the fixed charges on the bead surface, resulting in the negative sign of *τ*, which is like the temperature–permittivity relationship of a bulk liquid. However, on the droplet surface, mobile charges can move around to form dipole–charge complexes with water molecules regardless of environment, thus providing a dipole-oriented interface. The external heat perturbs both charges and solvent molecules and, consequently, an overall dipole orientation becomes randomized at the hot region, leading to the thermophilic behavior of droplets. As depicted in Fig. 3c for the 50% EtOH case, these interfacial charges are destabilized due to their weak interactions with EtOH and a lack of a homogeneous, tightly bound hydrogen bonding network, leading to the large permittivity gradient over temperature. On the other hand, DMSO can form strong complexes with water^41,42^, which is also evidenced by their highly exothermic mixing. This strong interaction leads to a highly ordered dipole alignment around the charged bead surface where the thermal stimulus indues the entropy-driven permittivity gradient. In contact with the PFP droplet, however, DMSO and water molecules construct a rigid interface with mobile charges optimally distributed to have a much strongly bound network, leading to the weak responsiveness of the droplet to a thermal field due to the inhibited mobility of the charges. We conclude that, rather than specific adsorption of negative charges on a PFP droplet, the interfacial charging can be attributed to the formation of mobile-charge–solvent networks, which spontaneously occurs to hydrate a hydrophobic surface.

## Discussion

The identity of negative ion species that are responsible for interfacial charging is still in question. Among autoionized species (H_3_O^+^ and OH^−^), OH^−^ might be a favorable network component as it has a more π-hydrogen bonding character than H_3_O^+^, which further strengthens the charge–dipole network through hydrogen bonds to hydrogens of water in addition to the charge–dipole electrostatic interaction. It has been revealed that OH^−^ (one O site) accepts more than three H-bonds from water while H_3_O^+^ (three H sites) donates three H-bonds to water (c.f., the total coordination number of three in the ideal tetrahedral coordination for the O site of OH^−^ and H sites of H_3_O^+^)^43–45^. This implies that OH^−^ accommodates more water molecules than H_3_O^+^ does upon hydration, and we attribute this hypercoordination of hydroxide ions with water to the π-hydrogen bonding characteristic of OH^−^. The concept of amphiphilicity of OH^−^ and H_3_O^+^ can be also considered^43^. The O site of OH^−^, which is hypercoordinated with water, can be regarded as a hydrophilic part with the H site of OH^−^ as a hydrophobic part. For H_3_O^+^, three H sites and one O site correspond to hydrophilic and hydrophobic parts, respectively. Taken altogether, OH^−^ accommodates more water molecules than H_3_O^+^ and resultant hypercoordination makes OH^−^ preferential amphiphilic species that occupy water/oil interfaces.

Another possibility for the negative interface comes with bicarbonate ions (HCO_3_^−^). Atmospheric carbon dioxide dissolves in water, producing carbonate ion species, mostly HCO_3_^−^ below pH 7. The critical contribution of HCO_3_^−^ to the charging of surfactant-free oil-in-water emulsions has been reported^46,47^. Given the oxygen-rich resonance structure of HCO_3_^−^, it is a good candidate for the ionic amphiphile at the interface. The hypercoordination of water molecules to the O sites of HCO_3_^−^ is predicted due to its π-bonding characteristic, thus having a highly hydrophilic oxygen-rich region with a relatively hydrophobic H site that points toward the oil phase. It is worth noting that perfluorocarbon liquid has a significantly high gas solubility than other classes of liquid. Even though our system contains only 1 vol% of PFP, the reaction of CO_2_ contained in PFP with water can occur at the interface during emulsification, which results in the interfacial enrichment of HCO_3_^−^.

It is also noteworthy that surface-active impurities could be responsible for interfacial charging. A possibility that fatty acids in hydrocarbon oils behave as surfactants at water/oil interfaces was suggested^11^ although there have been debates on it^48–50^. To examine the impurity effect, we conducted a comparison study in which distilled solvents were used. Most surface-active species would be charged species like acids. The boiling point of acids, when compared to that of organic compounds or perfluorocarbons with the same number of carbons, is much higher (e.g., 118 °C for acetic acid vs. 78 °C for ethanol and 140 °C for perfluoropentanoic acid vs. 29 °C for perfluoropentane). Thus, we rationalized that any acid impurities would be remarkably removed by distillation. Moreover, as perfluorocarbons are more chemically and thermally stable than hydrocarbon counterparts, it is less likely that impurities are generated in perfluorocarbon upon storage. PFP, ethanol and DMSO (vacuum applied) were distilled, and the first quarter of distillate was used. As shown in Supplementary Fig. 12, there is no distinct change in the drift velocity with the distilled solvents. If impurities were the major source of the interfacial charge, the reduced concentration of impurities in the distilled solvents would have affected the thermophoretic velocity to certain extent. Therefore, the arrangement of ionic species and water molecules might be the major sources of the interfacial charging and opto-thermophoresis although it would be practically impossible to prepare perfectly clean interfaces.

Some of the previous spectroscopic studies concluded that the interfacial charging arose from the asymmetry or imbalance of hydrogen bonds and the charge transfer from hydrogen bond acceptors to donors without inclusion of autoionized species^9,10^. However, the addition of DMSO might enhance the difference between hydrogen bond acceptors and donors as DMSO only serves as acceptors and result in less interfacial charges, which is inconsistent with our results on the DMSO–water binary mixture. Therefore, according to our studies, the presence of mobile negative charges and their strong anchoring with water molecules by cooperative electrostatic and hydrogen bonding interactions is a more likely scenario for the hydration of oil droplets, which is also suggested in a tetraphenylborate–water model^13^. Moreover, the hydrogen atoms of water in dangling hydrogen bonds, which point toward hydrophobic surfaces^16,51,52^, can be attributed to the existence of mobile negative charges near the droplet surfaces as proposed in our model.

To further investigate the scenario with OH^−^, molecular dynamics simulations for the density profile of OH^−^ at interfaces between PFP and three different aqueous phases were carried out (see Supplementary Note 3). As shown in Supplementary Fig. 13, OH^−^ ions are distributed near the PFP phase in all three systems. The relative peak density of OH^−^ is 50% DMSO > water > 50% EtOH and a relatively diffuse distribution of OH^−^ is observed with 50% EtOH, implying a stronger interfacial confinement of OH^−^ at the PFP/50% DMSO and a weaker confinement of OH^−^ in PFP/50% EtOH. The degree of the interfacial OH^−^ confinement can be correlated with the stabilization strength of OH^−^ by solvent molecules and furthermore the rigidness of interface made of ion-solvent complexes. These results are consistent with our opto-thermophoresis-based interpretations of the relationship between the thermal response of permittivity and interfacial structures.

On the other hand, the interfacial tension at liquid-liquid interfaces can be considered to understand the interfacial charging phenomenon. The interfacial tension between water and PFP is reported as 53.9 mN/m at 293.15 K^53^. The interfacial tension for PFP/50% EtOH and PFP/50% DMSO was estimated from the following equations that account for dispersion and polar components of surface tension (*γ*^d^ and *γ*^p^, respectively)^54,55^, ${\gamma }_{{{{{{\rm{A}}}}}}/{{{{{\rm{B}}}}}}}={\gamma }_{{{{{{\rm{A}}}}}}}+{\gamma }_{{{{{{\rm{B}}}}}}}-2\sqrt{{\gamma }_{{{{{{\rm{A}}}}}}}^{{{{{{\rm{d}}}}}}}{\gamma }_{{{{{{\rm{B}}}}}}}^{{{{{{\rm{d}}}}}}}}-2\sqrt{{\gamma }_{{{{{{\rm{A}}}}}}}^{{{{{{\rm{p}}}}}}}{\gamma }_{{{{{{\rm{B}}}}}}}^{{{{{{\rm{p}}}}}}}}$ and ${\gamma }_{{{{{{\rm{A}}}}}}}={\gamma }_{{{{{{\rm{A}}}}}}}^{{{{{{\rm{d}}}}}}}+{\gamma }_{{{{{{\rm{A}}}}}}}^{{{{{{\rm{p}}}}}}}$, where ${\gamma }_{{{{{{\rm{A}}}}}}/{{{{{\rm{B}}}}}}},{\gamma }_{{{{{{\rm{A}}}}}}},$and ${\gamma }_{{{{{{\rm{B}}}}}}}$ are interfacial tension between A and B, surface tension of A and B, respectively (see Supplementary Table 3 for details of calculated values). The obtained interfacial tensions for PFP/50% EtOH and PFP/50% DMSO are 16.9 and 31.7 mN/m, respectively. The interfacial tension can be regarded as a driving force for the interfacial accumulation of surfactant-like species^56^, which are the complexes of negative ions and solvent molecules in our systems. The remarkedly low interface tension between PFP and 50% EtOH implies less accumulation of the interfacial species. Along with the microscopic phase segregation of EtOH and water, this may contribute to the diffuse and fragile interface that results in the poor colloidal stability, low zeta potential, and strong opto-thermophoresis. The PFP/50% DMSO interface exhibits relatively high interfacial tension (which is still lower than that of PFP/water), thus promoting ions and molecules to be accumulated at the interface. Upon the accumulation, strong DMSO−water complexes can be locally formulated around the droplet, which strengthen the interfacial structure (i.e., rigid interface). We attribute this to the high colloidal stability, high zeta potential, and low thermal sensitivity of droplets in water-DMSO than in water, despite the calculated lower interfacial tension for PFP/50% DMSO. The effect of microscopic interactions between solvent molecules, especially between water and DMSO, could be more predominant in the hydration of microscale droplets than in bulk contact. Therefore, the actual interfacial tension for microscale droplets is predicted to be lowered, compared to the above-obtained values for the bulk contact.

In summary, probing the oil-water interfaces under a thermal field using OTTs provides a complementary perspective, in terms of thermal responsiveness of the interfaces and subsequent dipole arrangement of water and charge, on the long-standing mystery of water’s interfacial charging phenomena. Our analyses on the thermophoretic behavior of oil droplets in the different solvent compositions suggest that water and mobile ion species form interfacial layers at specific dipole arrangement to stabilize the hydrophobic matters. These opto-thermophoretic analyses can be extended to other oil materials such as n-alkanes and more polar oils to study contributions of oils to interfacial structures (e.g., the effects of their polarity and intermolecular interactions)^3^. This work is limited in directly identifying ionic species and their complexation with water, thus calling for the further studies in which opto-thermophoresis is combined with spectroscopic and computational techniques to elucidate the phenomena pertinent to interfacial behaviors of water. In addition, the capability of manipulating, assembling, and merging droplets in liquids will allow a wider range of applications of OTTs, including controllable microreactors^57,58^ and reconfigurable microfluidics^59^.

## Methods

### Materials and sample preparation

Deionized water was obtained from a Milli-Q water purification system (Millipore, Bedford, MA, USA). The pH and conductivity of water in the experiments were measured as ~6.7 and ~0.2 µS/cm, respectively. Perfluoropentane (PFP, 99.0%, FluoroMed, L.P.), ethanol (EtOH, ≥99.5%, Millipore Sigma) and dimethyl sulfoxide (DMSO, 99.9%, Fisher Chemical) were used as received. Carboxylate-functionalized polystyrene (PS) beads (2.19 µm, cross-linked, 2% divinylbenzene) were purchased from Bangs Laboratories, Inc. Surfactant-free PFP-in-water emulsions were prepared by sonicating a mixture of PFP and water (1:99, v/v) in a bath sonicator (Branson 2800, 40 kHz) for 10 sec^60^. Emulsions in binary solvents (50 wt%) were prepared at the same volume ratio. Gold nanoislanlds (Au NIs) substrates were prepared by thermally depositing 4.5 nm gold thin films on glass slides (Denton thermal evaporator, bar pressure: 1 × 10^−5 ^Torr) followed by thermal annealing in a box furnace (Lindberg Blue M, Thermo Fischer Scientific) at 550 °C for 2 h^61^.

The zeta potential of emulsions was recorded by the dynamic light scattering (DLS) method with a 633 nm laser in a backscattering mode (Zetasizer Nano ZS and Zetasizer software v7.18, Malvern Instruments). Samples were loaded into a folded capillary cell, and the temperature was set at 20 °C (120 min of equilibration time). Henry’s function $f\left(\kappa a\right) \sim 1.3$ (${\kappa }^{-1}$: Debye length and $a$: particle radius) was used. For the binary solvent mixtures, the following dispersant properties were used: 2.41 mPa s (viscosity), 1.362 (refractive index), 50.3 (dielectric constant) for 50% EtOH, and 3.46 mPa s (viscosity), 1.407 (refractive index), 75.5 (dielectric constant) for 50% DMSO.

### Optical setup

An inverted microscope (Nikon Ti-E) with a 100x oil objective (NA 1.3, Nikon) was used. A 532 nm laser (Genesis MX STM-1 W, Coherent) beam was expanded by a 5x beam expander (GBE05-A, Thorlabs) and focused onto a substrate stage. A notch filter (533 nm) was placed between the objective and CCD to block the incident laser beam and images were obtained through a color charge-coupled device (CCD, Nikon). White light was directed from the top of the stage for bright-field imaging. Emulsion samples were loaded onto the plasmonic substrate with a cylindrical spacer (100 μm in height), which was covered by a cover slip to minimize the evaporation of solvent during experiments (Supplementary Fig. 1).

### Measurement of trapping velocity

Trapping of a single PFP droplet was recorded with a CCD camera (100 ms exposure time). The trajectory of the droplet was measured at each frame (1 frame = 0.1 s) and the velocity was derived from a linear region of the trajectory (Supplementary Fig. 7).

### Temperature measurements

Microscale temperature profiles were obtained through thermal imaging by quadriwave shearing interferometry (TIQSI)^62^. A thermal imaging camera (SID4-HR, Phasics) was coupled to an inverted microscope (Nikon Ti-E) with a 100x oil objective (NA 1.3, Nikon). Water was used as a medium and thermal imaging was recorded through a SIDFTHERMO software (Phasics). Since it was not possible to directly obtain the temperature profile at low optical power used in the trapping studies, temperatures at higher optical power were first measured (Supplementary Fig. 8), then the temperature profile at the working power was calculated using linear extrapolation from a maximum temperature–optical power curve with the fixed width from measurements (Supplementary Fig. 9).

### Simulation of temperature profiles

A finite-element solver (COMSOL Multiphysics) was used to simulate temperature profiles. Cross-sectional geometry of a 10 μm × 10 μm water domain was constructed. Gaussian heat influx (~*A* exp [−2*r*^2^*/ω*^2^]) was set to coincide with the derived temperature profile from Supplementary Fig. 9 and coupled to heat transfer in water. For binary solvents (50% EtOH and DMSO), temperature-dependent functions of heat capacity at constant pressure, *C*_*p*_ and thermal conductivity, *κ* were derived from literature^63–65^ (Supplementary Table 1) and used along with the fixed heat influx obtained in water.

## Supplementary information

Supplementary Information

Description of Additional Supplementary Files

Supplementary Movie 1

Supplementary Movie 2

## Data Availability

The data supporting the findings of this study are available within the article and its Supplementary Information files as well as Source Data. Any other data relevant to this study are available from the corresponding author upon request. Source data are provided with this paper.

## Source data

Source Data
