# Supplementary material for: Investigating water/oil interfaces with opto-thermophoresis
Source: Nat Commun. 2022 Jun 29;13:3742. doi: 10.1038/s41467-022-31546-3 (PMC9243056; doi:10.1038/s41467-022-31546-3)
Supplement: Supplementary file 1 — Supplementary Information [file 41467_2022_31546_MOESM1_ESM.pdf]

Supplementary Information

Investigating water/oil interfaces with opto-thermophoresis

Kim et al.

Supplementary Figures 1–13

Supplementary Tables 1–3

Supplementary Notes 1–3

Supplementary References

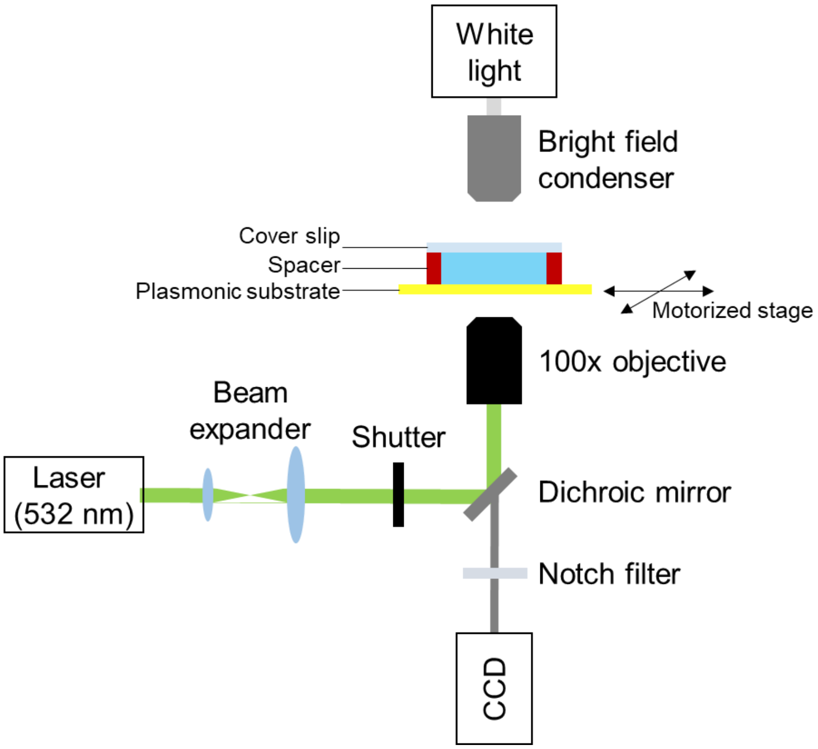

Supplementary Fig. 1: Optical setup for OTTs with in situ optical microscopy.

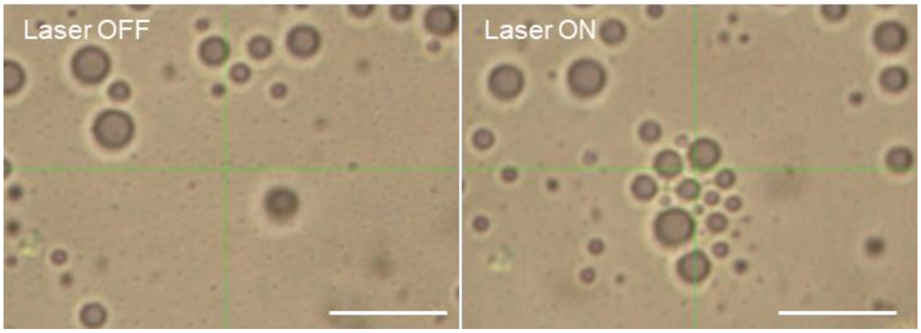

Supplementary Fig. 2: Optical images of many PFP droplets trapped by OTTs in a concentrated sample (scale bars: 10 µm).

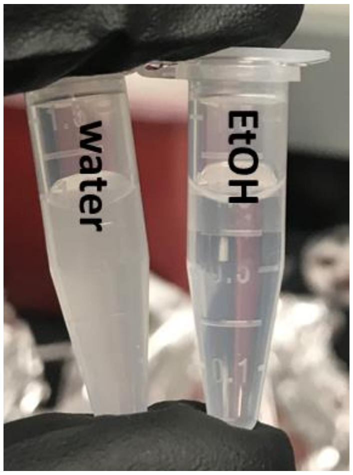

Supplementary Fig. 3: **Photograph of emulsions prepared in water and EtOH.**

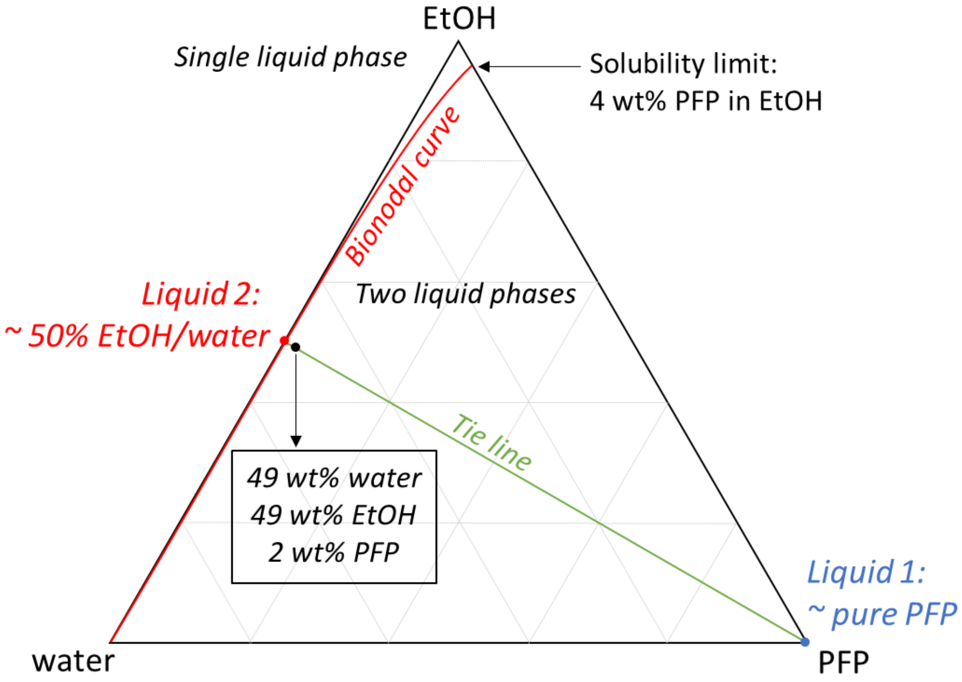

Supplementary Fig. 4: Phase diagram of a ternary liquid system (water + EtOH + PFP).

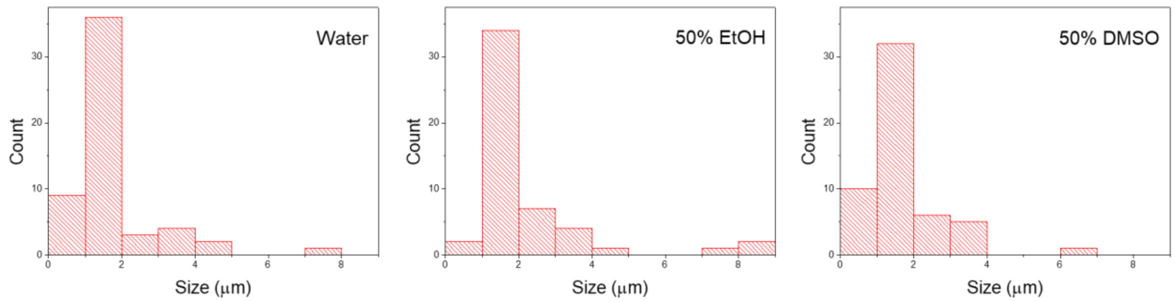

Supplementary Fig. 5: Size distributions of PFP droplets prepared in water, 50% EtOH, and 50% DMSO.

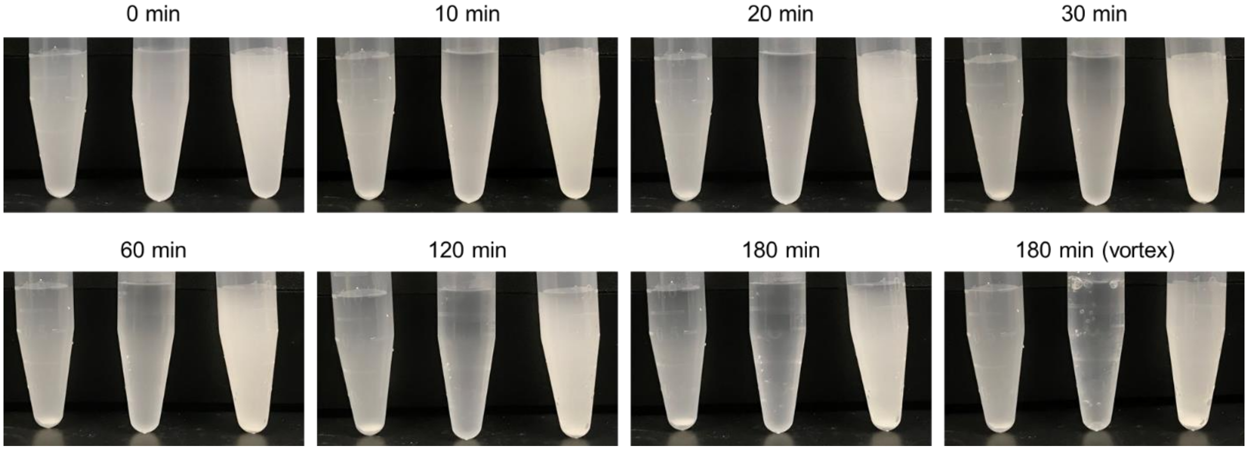

Supplementary Fig. 6: Photographs of emulsions prepared in the different solvents (from left to right: water, 50% EtOH, and 50% DMSO) stored at room temperature. The final photograph was taken after brief mixing to better show the presence of emulsions in the water sample.

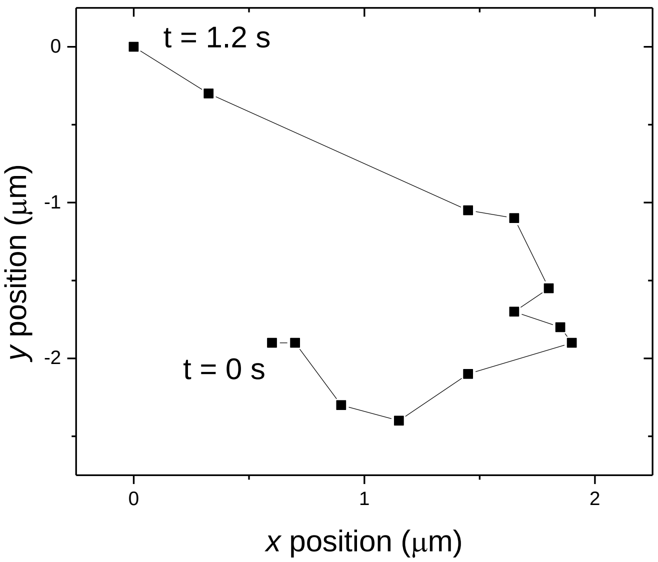

Supplementary Fig. 7: Trajectory of a single PFP droplet measured at each frame (1 frame = 0.1 s). A trapping velocity was calculated from a linear portion of the trajectory, mostly for the last 1-2 frames.

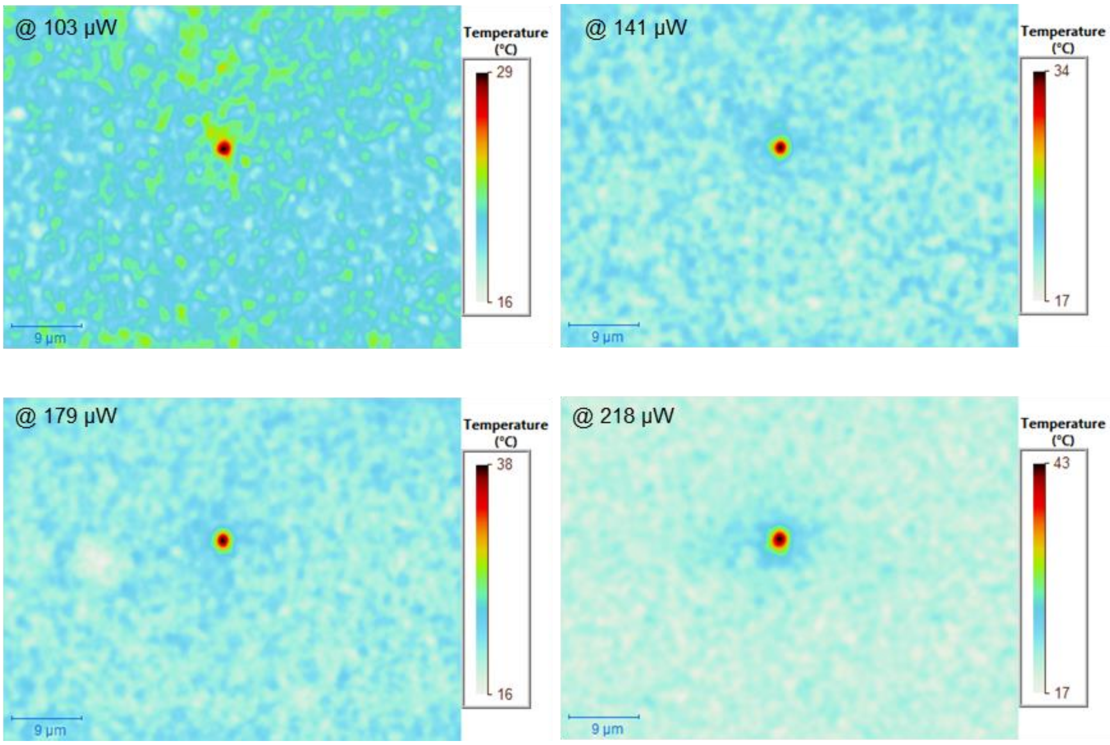

Supplementary Fig. 8: **Temperature distribution maps of a plasmonic substrate around a laser beam at varying optical power measured by thermal imaging.**

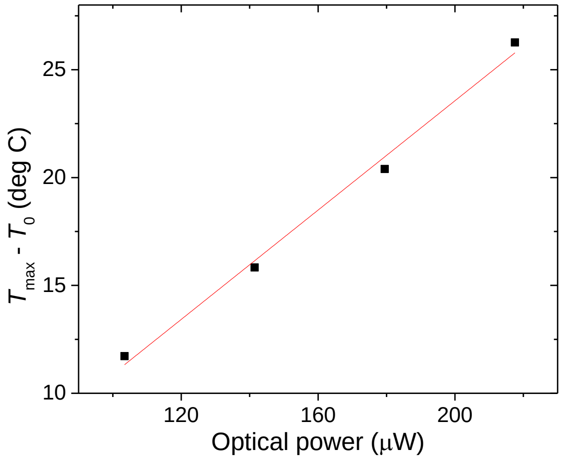

Supplementary Fig. 9: Dependence of maximum temperature on optical power. A linear correlation enables the derivation of working temperature in trapping experiments by extrapolation.

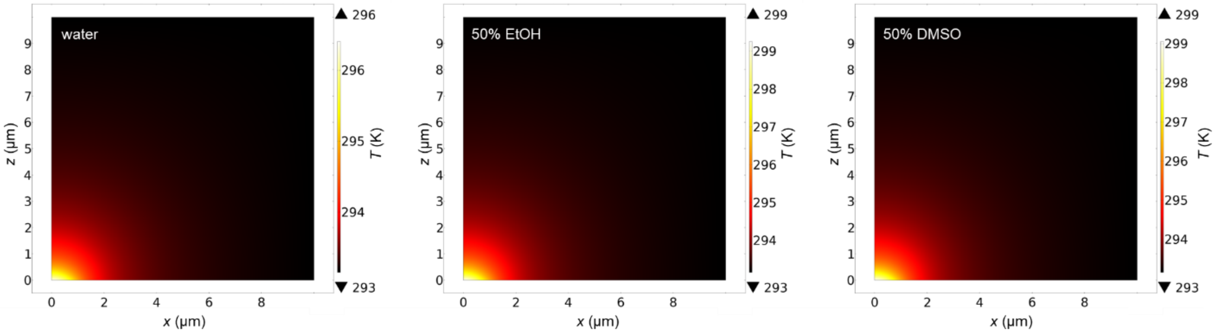

Supplementary Fig. 10: **Simulated temperature profiles in different solvents.**

Supplementary Table 1: Temperature dependence of heat capacity at constant pressure, C_p, and thermal conductivity, κ, of solvents used in the simulation of temperature profiles. Representing C_p or κ, polynomial functions $f(T)$ with coefficients A_N for the Nth power of temperature T were obtained by fitting literature data[1–3].

		$f(T) = A_0 + A_1T + A_2T^2 + ...$				
		A_0	A_1	A_2	A_3	A_4
water	C_p [J kg^{-1} K^{-1}]	1.20E+03	-8.04E+01	3.10E-01	-5.38E-04	3.62E-07
	κ [W m^{-1} K^{-1}]	-8.69E-01	8.95E-03	-1.58E-05	7.98E-09	
50% EtOH	C_p [J kg^{-1} K^{-1}]	-6.27E+03	6.06E+01	-8.93E-02		
	κ [W m^{-1} K^{-1}]	1.83E-01	6.69E-04	-7.14E-07	1.12E-19	
50% DMSO	C_p [J kg^{-1} K^{-1}]	-2.39E+03	2.83E+01	-3.32E-02		
	κ [W m^{-1} K^{-1}]	-4.79E-02	2.10E-03	-3.09E-06	1.00E-09	

Supplementary Note 1. Effect of dispersion force in opto-thermophoresis

The van der Waals interaction between solvents and colloids affects thermophoresis. As the solvent expands upon heating, solvent in a cold region has a higher density than a hot region, resulting in a stronger van der Waals interaction. This effect induces a slip flow that drives colloids from hot to cold (i.e., thermophobic), termed as dispersion force[4,5]. The drift velocity by the dispersion force under a temperature gradient is formulated as, $\mathbf{u} = -\frac{2\beta H_{12}}{9\pi d_0}\nabla T$ where β is the thermal expansivity of solvent, H_{12} is the Hamaker constant of colloid-solvent interactions, and d_0 is the molecular length scale of solvent[4]. The Hamaker constant represents the van der Waals interaction between to materials, which is related to the dielectric response function $\varepsilon''(\omega)$ for the range of frequencies from the infrared to the far ultraviolet[6,7], as mentioned by the reviewer. However, lack of the available full $\varepsilon''(\omega)$ spectrum for all the materials used in our study makes it difficult to calculate the Hamaker constant from $\varepsilon''(\omega)$. Instead, we used the following approximate equation to obtain the Hamaker constant from the static dielectric constant ε and refractive index n[6,8].

$$H_{12} \approx \frac{3}{4}k_{\mathrm{B}}T\left(\frac{\varepsilon_1 - \varepsilon_2}{\varepsilon_1 + \varepsilon_2}\right)^2 + \frac{3h\nu_{\mathrm{e}}}{16\sqrt{2}}\frac{(n_1^2 - n_2^2)^2}{(n_1^2 + n_2^2)^{3/2}}$$

where ν_{e} is the main electronic absorption frequency in the UV region and assumed to be the same for all the materials (3×10^{15} Hz, plasma frequency)[6,8]. The subscript numbers 1 and 2 correspond to solvent and colloid, respectively. The obtained H for three system and calculated dispersion force-driven drift velocity is shown in Table S2 along with other physicochemical properties the materials used in the calculations. The positive sign of the calculated velocity indicates that the dispersion force drives an outward movement of droplets from a heat source. In comparison to the observed velocities, the contribution of the dispersion force to the opto-thermophoresis is shown to be little. Therefore, we ascribe the observed thermophilic movement of droplets mainly to the permittivity-temperature gradient.

Supplementary Table 2: Calculation of the Hamaker constant and drift velocity by dispersion force. ε and n of PFP are 1.75 and 1.24, and d_0 is fixed at 10^{-9} m.

	ε	n	H_{12} [J]	η [Pa s]	β [K^{-1}]	∇T [K m^{-1}]	**u** [μm s^{-1}]	$\mathbf{u}_{obs}$ [μm s^{-1}]
water	80.1	1.33	5.13E-21	1.00E-03	2.07E-04	-1.99E+06	0.15	-7.6
50% EtOH	50.0	1.36	6.76E-21	2.41E-03	8.45E-04	-3.74E+06	0.63	-20.9
50% DMSO	75.5	1.41	1.08E-20	3.46E-03	6.11E-04	-3.61E+06	0.49	-3.7

Supplementary Note 2. Marangoni effect in opto-thermophoresis

An interfacial tension gradient induced by the temperature gradient in opto-thermophoresis setting can also affect the behavior of droplets. This is so-called the Marangoni effect at fluid-fluid interfaces. The flow dynamics including Marangoni flow and convective flow at water/oil interfaces was studied using computational simulation.

A finite-element solver (COMSOL Multiphysics) was used to simulate flow profiles. Three-dimensional geometry was constructed with a spherical PFP domain (2 μm in diameter at 2 μm distance from the origin) within a cylindrical solvent domain (20 μm base diameter × 10 μm height), enclosed with solid boundaries on all sides. Gaussian heat influx ($\sim \exp\,[-2r^2/\omega^2]$), obtained from the measured temperature profile in Supplementary Figs. 7 and 8, was set at the origin and coupled to heat transfer in liquid. The heat transfer module and the laminar modules were coupled to model Marangoni and Rayleigh Bernard convection throughout the domains. Marangoni flow in the simulation domains was induced by implementing an interfacial tension gradient along the PFP/solvent interface as a function of temperature (modelled as a slip interior wall) while nonslip walls for other boundaries. The function of interfacial tension with respect to temperature was derived from literature data[9–11].

The temperature-driven interfacial tension gradient along the droplet surface generates the outward slip flow of a surrounding medium, subsequently resulting in the thermophilic movement of the droplet (Supplementary Fig. S10). Net velocities by Marangoni flow were obtained as −1.0, −1.5, −1.6 μm/s in water, 50% EtOH, and 50% DMSO, respectively. These results indicate that the interfacial tension gradient induced by the temperature gradient contributes to the opto-thermophoresis of oil droplets, but the permittivity gradient is a major factor that explains the observed drift velocities that vary significantly by solvents.

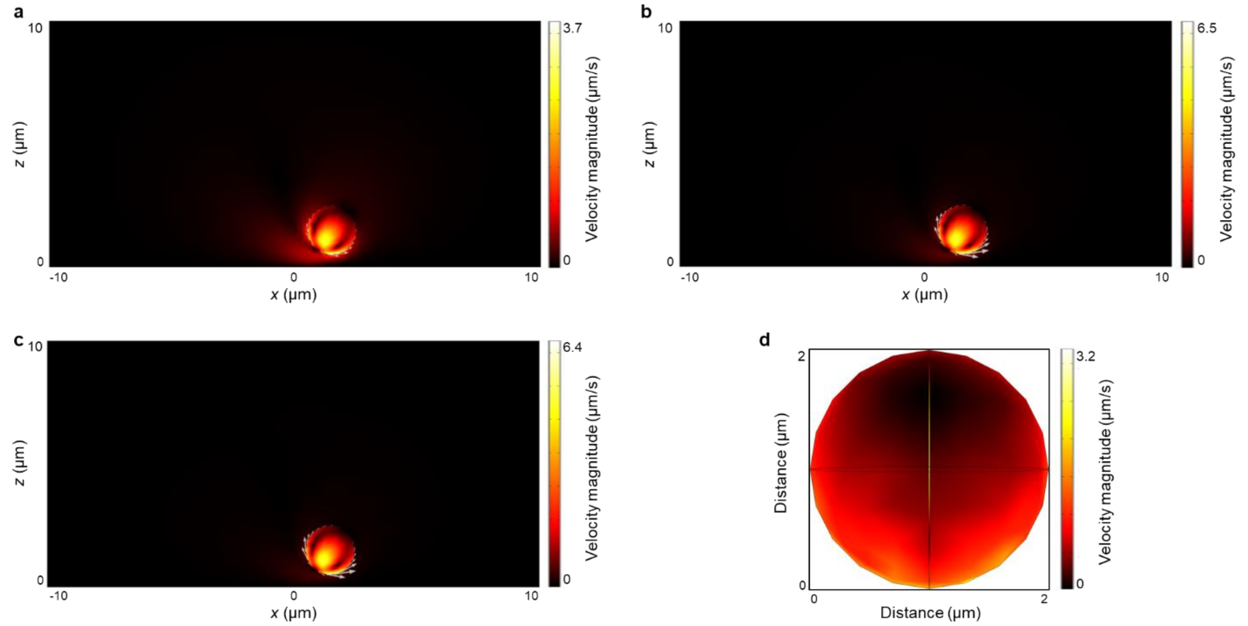

Supplementary Fig. 11: Simulated flow profiles of PFP droplets in different solvents (vertical cross sections). (a) water, (b) 50% EtOH, (c) 50% DMSO, and (d) velocity map at the PFP/water interface. Arrows in (a-c) indicate Marangoni flow at interfaces, which is much stronger than Rayleigh–Bénard convection flow throughout the domain.

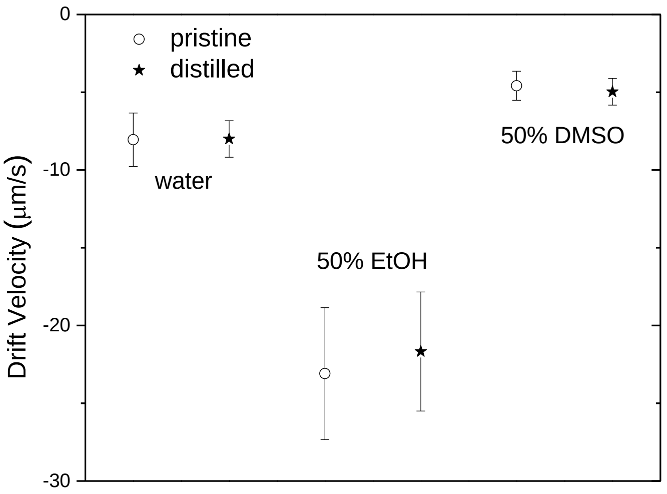

Supplementary Fig. 12: Drift velocity of PFP droplets in pristine and distilled solvents (mean ± standard deviation, n = 10).

Supplementary Note 3. Molecular dynamics simulation

All MD simulations were carried out with the LAMMPS package[12]. The four-site transferrable intermolecular potential (TIP4P)/2005 model was used for water[13]. The force fields of PFP molecule, hydroxide anion, ethanol molecule, and DMSO molecule were taken from Watkins et al[14], Vácha et al[15], Mijaković et al[16]. The potential energy equation is based on the assisted model building with energy refinement (AMBER) potential[17]:

$$E_{\text{total}} = \sum_{\text{bonds}} K_r(r - r_0)^2 + \sum_{\text{angles}} K_\theta(\theta - \theta_0)^2 + \sum_{\text{torsions}} \frac{K_\phi}{2}(1 + \cos(n\phi - \gamma)) + \sum_{i=1}^{N} \sum_{j=i+1}^{N} \left\{4\epsilon_{ij}\left[\left(\frac{\sigma_{ij}}{r_{ij}}\right)^{12} - \left(\frac{\sigma_{ij}}{r_{ij}}\right)^{6}\right] + \frac{q_i q_j}{r_{ij}}\right\}$$

where K_r, K_θ, and K_ϕ are the bond, angle, and torsion constants; r, θ, and ϕ are the bond lengths, bond angles, and torsion angles; r_0, θ_0, and γ are the equilibrium bond lengths, bond angles, and torsion angles; ϵ_{ij} and σ_{ij} are the Lennard-Jones potential parameters; r_{ij} is the separation between atoms i and j; and q_i andq_j are the charges of atoms. The Lorentz-Berthelot mixing rules were used for the mixed ϵ_{ij} and σ_{ij} parameters, respectively. The velocity Verlet algorithm is employed in integrating equations of motion, and the time step is 1 fs. Initially, the isothermal–isobaric (NPT) ensemble is employed to reach the required temperature (293 K) and pressure (1 atm). Then, the system is equilibrated under the canonical ensemble (NVT) with the Nose-Hoover heat reservoir at the target temperature for 0.5 ps, followed by relaxation under a microcanonical ensemble (NVE) for 0.2 ns. Finally, a production step of 2 ns was adopted under the NVE condition, during which the ion and molecule densities were calculated every 10 fs. For each case, four independent simulations were performed with different initial atom velocity assignments, implemented by using different seeds for random number generation. Averaged values were obtained to improve the reliability of the simulation results. The SHAKE algorithm[18] was employed to fix geometries of the water molecules and hydroxide ions. Long range electrostatic interactions were counted using the particle-particle particle-mesh (PPPM) method[19] with a precision of 10^{-6}.

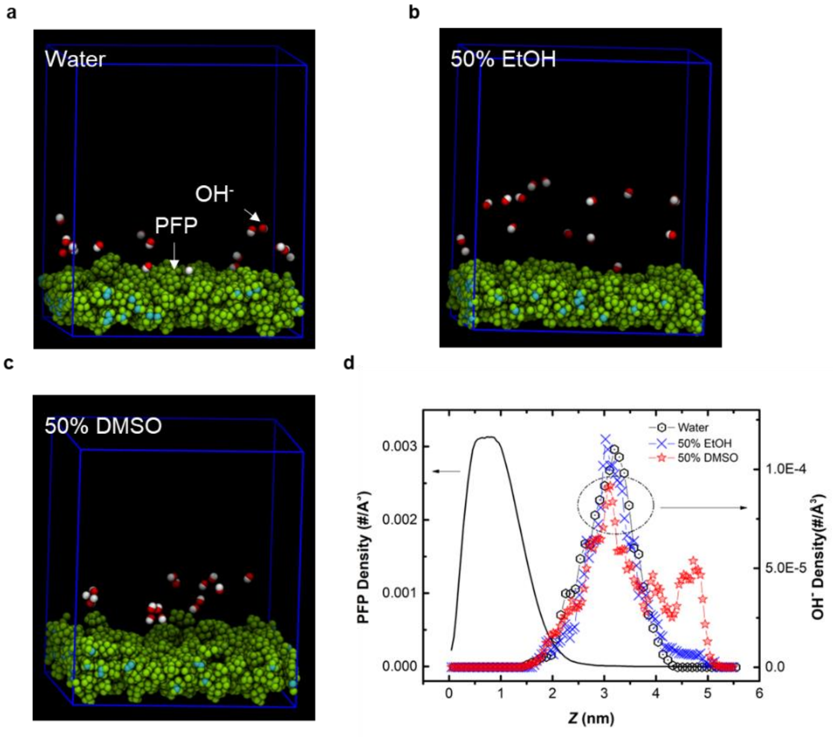

Supplementary Fig. 13: Molecular Dynamics simulation of OH^- density profile at interfaces of PFP with water, 50% ethanol, and 50% DMSO. (a-c) Snapshots of the three interfaces, in which aqueous solutions were not plotted for clarity. (d) Number density profiles of hydroxide anions at water/PFP (black), 50% ethanol/PFP (red), 50% DMSO/PFP interfaces (blue). Black solid line corresponds to the number density of PFP.

Supplementary Table 3: Calculation of the interfacial tension of PFP/50% EtOH and PFP /50% DMSO using dispersion and polar terms of surface tension (γ^d and γ^p, respectively). All the surface and interfacial tension data are in mN/m.

	water[20,21]	PFP[9]	silicone oil[22]	50% EtOH[23,24]	hexane[25]	50% DMSO[25,26]	PFP/ 50% EtOH	PFP/ 50% DMSO
γ	72.8	10.1	21.1	28.5	17.9	57.6		
γ^d	21.8	10.1	19.6	11.7	17.9	32.0		
γ^p	51.0	0	1.5	16.8	0	25.5		
$\gamma_{interface}$							16.9	31.7

Supplementary References

1. Melinder, Å. Thermophysical properties of aqueous solutions used as secondary working fluids. (KTH Royal Institute of Technology, 2007).
2. Checoni, R. F. & Volpe, P. L. O. Measurements of the molar heat capacities and excess molar heat capacities for water + organic solvents mixtures at 288.15 K to 303.15 K and atmospheric pressure. *J. Solution Chem.* **39**, 259–276 (2010).
3. Zhou, J.-C., Che, Y.-Y., Wu, K.-J., Shen, J. & He, C.-H. Thermal conductivity of DMSO + C_2H_5OH, DMSO + H_2O, and DMSO + C_2H_5OH + H_2O mixtures at T = (278.15 to 338.15) K. *J. Chem. Eng. Data* **58**, 663–670 (2013).
4. Würger, A. Thermal non-equilibrium transport in colloids. *Reports Prog. Phys.* **73**, (2010).
5. Liu, S., Lin, L. & Sun, H. B. Opto-thermophoretic manipulation. *ACS Nano* **15**, 5925–5943 (2021).
6. Butt, H.-J. & Kappl, M. *Surface and Interfacial Forces*. (Wiley-VCH, 2010).
7. Fernández-Varea, J. M. & Garcia-Molina, R. Hamaker constants of systems involving water obtained from a dielectric function that fulfills the f sum rule. *J. Colloid Interface Sci.* **231**, 394–397 (2000).
8. Israelachvili, J. N. *Intermolecular and Surface Forces*. (Elsevier, 2011).
9. Morgado, P., Gaspar, J. & Filipe, E. J. M. Liquid–liquid interfaces: Water–perfluoroalkanes and water–perfluoroalkylalkanes, experimental interfacial tensions and molecular simulation. *J. Mol. Liq.* **312**, 113385 (2020).
10. Makkonen, L. & Kurkela, J. Another look at the interfacial interaction parameter. *J. Colloid Interface Sci.* **529**, 243–246 (2018).
11. Jańczuk, B. *et al.* The usefulness of the equation of state for interfacial tensions estimation in some liquid-liquid and solid-liquid systems. *J. Colloid Interface Sci.* **181**, 108–117 (1996).
12. Plimpton, S. Fast parallel algorithms for short-range molecular dynamics. *J. Comput. Phys.* **117**, 1–19 (1995).
13. Abascal, J. L. F. & Vega, C. A general purpose model for the condensed phases of water: TIP4P/2005. *J. Chem. Phys.* **123**, 234505 (2005).
14. Watkins, E. K. & Jorgensen, W. L. Perfluoroalkanes: Conformational analysis and liquid-state properties from ab initio and Monte Carlo calculations. *J. Phys. Chem. A* **105**, 4118–4125 (2001).

15. Vácha, R., Megyes, T., Bakó, I., Pusztai, L. & Jungwirth, P. Benchmarking polarizable molecular dynamics simulations of aqueous sodium hydroxide by diffraction measurements. *J. Phys. Chem. A* **113**, 4022–4027 (2009).
16. Mijaković, M. *et al.* A comparison of force fields for ethanol–water mixtures. *Mol. Simul.* **41**, 699–712 (2015).
17. Cornell, W. D. *et al.* A second generation force field for the simulation of proteins, nucleic acids, and organic molecules. *J. Am. Chem. Soc.* **117**, 5179–5197 (1995).
18. Hockney, R. . & Eastwood, J. *Computer Simulation Using Particles*. (CRC Press, 2021).
19. Sun, H. COMPASS: An ab initio force-field optimized for condensed-phase applications - Overview with details on alkane and benzene compounds. *J. Phys. Chem. B* **102**, 7338–7364 (1998).
20. Binks, B. P. & Clint, J. H. Solid wettability from surface energy components: Relevance to pickering emulsions. *Langmuir* **18**, 1270–1273 (2002).
21. Fowkes, F. M. Additivity of intermolecular forces at interfaces. I. Determination of the contribution to surface and interfacial tensions of dispersion forces in various liquids 1. *J. Phys. Chem.* **67**, 2538–2541 (1963).
22. Peters, F. & Arabali, D. Interfacial tension between oil and water measured with a modified contour method. *Colloids Surfaces A Physicochem. Eng. Asp.* **426**, 1–5 (2013).
23. Sohounhloue, D. *et al.* Thermodynamic properties and structure of the water–ethanol silicone oil interface. *Can. J. Chem.* **65**, 2299–2304 (1987).
24. Vazquez, G., Alvarez, E. & Navaza, J. M. Surface tension of alcohol + water from 20 to 50 °C. *J. Chem. Eng. Data* **40**, 611–614 (1995).
25. Schrader, A. M. *et al.* Correlating steric hydration forces with water dynamics through surface force and diffusion NMR measurements in a lipid-DMSO-H_2O system. *Proc. Natl. Acad. Sci. U. S. A.* **112**, 10708–10713 (2015).
26. Markarian, S. A. & Terzyan, A. M. Surface tension and refractive index of dialkylsulfoxide + water mixtures at several temperatures. *J. Chem. Eng. Data* **52**, 1704–1709 (2007).
