# Supplementary material for: Investigating water/oil interfaces with opto-thermophoresis
Source: Nat Commun. 2022 Jun 29;13:3742. doi: 10.1038/s41467-022-31546-3 (PMC9243056; doi:10.1038/s41467-022-31546-3)
Supplement: Supplementary file 2 — Description of Additional Supplementary Files [file 41467_2022_31546_MOESM2_ESM.pdf]

Description of Additional Supplementary Files

File Name: Supplementary Movie 1
Description: Trapping of a single PFP droplet by OTTs.

File Name: Supplementary Movie 2
Description: Manipulation of a single PFP droplet by OTTs.
